# Effects of Feedstock and Pyrolysis Temperature on Biochar Adsorption of Ammonium and Nitrate

**DOI:** 10.1371/journal.pone.0113888

**Published:** 2014-12-03

**Authors:** Xiapu Gai, Hongyuan Wang, Jian Liu, Limei Zhai, Shen Liu, Tianzhi Ren, Hongbin Liu

**Affiliations:** 1 Key Laboratory of Nonpoint Source Pollution Control, Ministry of Agriculture/Institute of Agricultural Resources and Regional Planning, Chinese Academy of Agricultural Sciences, Beijing, China; 2 United States Department of Agriculture–Agricultural Research Service (USDA-ARS), Pasture Systems and Watershed Management Research Unit, University Park, Pennsylvania, United States of America; 3 Institute of Agro-Environmental Protection, Ministry of Agriculture, Tianjin, China; Glasgow University, United Kingdom

## Abstract

Biochar produced by pyrolysis of biomass can be used to counter nitrogen (N) pollution. The present study investigated the effects of feedstock and temperature on characteristics of biochars and their adsorption ability for ammonium N (NH_4_^+^-N) and nitrate N (NO_3_^−^-N). Twelve biochars were produced from wheat-straw (W-BC), corn-straw (C-BC) and peanut-shell (P-BC) at pyrolysis temperatures of 400, 500, 600 and 700°C. Biochar physical and chemical properties were determined and the biochars were used for N sorption experiments. The results showed that biochar yield and contents of N, hydrogen and oxygen decreased as pyrolysis temperature increased from 400°C to 700°C, whereas contents of ash, pH and carbon increased with greater pyrolysis temperature. All biochars could sorb substantial amounts of NH_4_^+^-N, and the sorption characteristics were well fitted to the Freundlich isotherm model. The ability of biochars to adsorb NH_4_^+^-N followed: C-BC>P-BC>W-BC, and the adsorption amount decreased with higher pyrolysis temperature. The ability of C-BC to sorb NH_4_^+^-N was the highest because it had the largest cation exchange capacity (CEC) among all biochars (e.g., C-BC400 with a CEC of 38.3 cmol kg^−1^ adsorbed 2.3 mg NH_4_^+^-N g^−1^ in solutions with 50 mg NH_4_^+^ L^−1^). Compared with NH_4_^+^-N, none of NO_3_^−^-N was adsorbed to biochars at different NO_3_^−^ concentrations. Instead, some NO_3_^−^-N was even released from the biochar materials. We conclude that biochars can be used under conditions where NH_4_^+^-N (or NH_3_) pollution is a concern, but further research is needed in terms of applying biochars to reduce NO_3_^−^-N pollution.

## Introduction

Today, biochar is receiving great research attention due to its potential importance in agronomic and environmental applications. Biochar refers to a carbon (C)-rich and porous substance, which is produced by thermal decomposition of biomass under oxygen-limited conditions and at relatively low temperatures (<700°C) [1]. It has a high specific surface area, a high density of negative surface charges, and characteristic pores and surface functional groups [2]. Biochar has been reported to be able to improve soil fertility by sequestrating C and enhancing retention of nutrients [2]–[5] and to suppress greenhouse gas emissions to the air [6].

Leaching of nitrogen (N) from agricultural land caused by excessive application of N fertilizers may pose a great threat to the quality of surface- and groundwater, and results in eutrophication of water bodies [7]. This is a particular concern in China that is consuming about one third of the total N fertilizers in the world [8]. Biochar is considered as a potential applicable material to mitigate N leaching, since a few studies have indicated that it can affect availability and cycling of N in the soil [9]–[12]. However, confounding results have been reported with regard to the effect of biochar application on N leaching. For example, Ding et al. [10] observed a reduction of NH_4_^+^-N leaching at 0.2-m soil depth by 15% and Laird et al. [11] observed a reduction of total N leaching by 11% in typical US Midwestern agricultural soils after addition of biochar to the surface soil layer. Based on these findings, they concluded that biochar, mainly owing to a high N sorption capacity, can be used as an effective soil amendment to reduce N losses from soils. However, some studies oppositely showed a limited or no ability of biochar to adsorb NO_3_^−^-N. For instance, Hollister et al. [13] observed no adsorption of NO_3_^−^ to biochar derived from corn (*Zea mays* L.) or oak (*Quercus* spp.). Yao et al. [14] found nine of thirteen biochars tested had little NO_3_^−^ adsorption ability and some even released NO_3_^−^ into water solution. These contradictory results are likely because of the differences in properties among the biochars, which poses an urgent need to disclose the relationship between biochar characteristics and their effects on adsorption of NH_4_^+^-N and NO_3_^−^-N.

Feedstock and temperature during pyrolysis can influence molecular structure and pores size distribution of biochar, and thus affect biochar sorption characteristics [15]–[16]. Sohi et al. [17] reported that different feedstocks resulted in different magnitudes of surface area, pores and functional groups in biochars, and all these variables affect sorption characteristics of biochars. Sun et al. [18] reported that poultry-litter biochar had a larger specific surface area and porosity than wheat-straw biochar, despite the two biochars were produced under the same temperature (400°C). In general, high pyrolysis temperature leads to greater specific surface area and aromaticity of biochar [15]. For example, charcoal made from wheat residue at 500–700°C is well carbonized and its specific surface area is relatively high (>300 m^2^ g^−1^), whereas chars formed at 300–400°C are partially carbonized and have a lower specific surface area (<200 m^2^ g^−1^) [19]. Moreover, low-temperature biochars (250–400°C) will probably be more suitable for improving soil fertility than high-temperature ones due to the relatively stable aromatic backbone from pyrolysis and more C = O and C-H functional groups which may serve as nutrient exchange sites after oxidation [20]. Addition of low-temperature biochars to soils was reported to improve soil fertility by raising soil cation exchange capacity (CEC) [2]. On the other hand, Gell et al. [21] reported that low temperature biochars were more phytotoxic due to accumulation of tars and other organic compounds. So temperature of pyrolysis plays a great role in biochar properties. Moreover, decrease of atomic ratios H/C and O/C resulted from removing H- and O-containing functional groups with increasing temperature will produce high aromaticity and low polarity biochars [22].

In recent years, development of new techniques has provided a great opportunity to better understand biochar components and structures. These new techniques include Fourier transform-infrared spectroscopy (FT-IR) and field emission-scanning electron microscopy (FE-SEM), which can be used to characterize the surface functional groups and micro-morphology of biochars. The main objectives of this study were to (i) investigate the effects of feedstock types and pyrolysis temperature on biochar characteristics related to N adsorption ability; and (ii) determine the main factors affecting the adsorption of NH_4_^+^-N and NO_3_^−^-N to biochars. These will help to gain insights in use of biochar to mitigate nonpoint source pollution from agricultural soils.

## Materials and Methods

### Preparation of biochars

Biochar samples were produced from three common agricultural by-products: wheat-straw, corn-straw and peanut-shell. Raw materials were cut into small pieces (2 cm) and oven-dried (70°C) for 2 days after washing with deionized (DI) water for five times. The materials were then ground and sieved to yield a uniform 1 mm size fraction, and converted to biochar under oxygen-limited conditions using a muffle furnace (SXZ-12-10). To minimize oxygen content at reaction, the container was filled with the feedstock materials and tightly sealed. The pyrolysis temperature was raised to the aimed values of 400°C, 500°C, 600°C and 700°C and held constant for 1.5 h [19]. Biochar yields were recorded and the resulting twelve biochar samples were hereafter referred as W-BC400, W-BC500, W-BC600, W-BC700, C-BC400, C-BC500, C-BC600, C-BC700, P-BC400, P-BC500, P-BC600 and P-BC700. The biochar production rate at each temperature was calculated as: Production rate (%)  =  (M_Biochar_/M_Feedstock_) ×100, where M_Biochar_ is the mass of biochar and M_Feedstock_ is the mass of feedstock, both on a basis of dry weight. Detailed information about the chemical and physical characteristics of biochars is listed in Table 1 and Table 2.

**Table 1:** The yields, chemical compositions and atomic ratios of biochars produced from different feedstocks at different pyrolytic temperatures.

Biochars	Temp. (°C)	Yield (%)	Component (%)				Atomic ratio			
			C	N	H	O	C:N	O:C	H:C	(O+N)/C
**W-BC**	400	32.4	57.8a	1.5b	3.2c	21.6c	44.2c	0.56c	0.66bc	0.22b
	500	27.6	70.3d	1.4ab	2.9bc	17.7b	57.8d	0.38ab	0.49b	0.15ab
	600	24.6	73.4e	1.4ab	2.1bc	14.9a	62.0de	0.31a	0.35ab	0.12a
	700	22.8	73.9e	1.2a	1.3a	14.6a	74.4e	0.30a	0.22a	0.12a
**C-BC**	400	35.5	56.1a	2.4e	4.3d	22.0c	27.9a	0.59c	0.92c	0.24b
	500	29.3	58.0ab	2.3d	2.7bc	21.5c	29.3a	0.57c	0.56b	0.22b
	600	26.7	58.6ab	2.0c	2.0b	18.7b	34.7b	0.48bc	0.41ab	0.19ab
	700	24.9	59.5b	1.6b	1.5a	16.6ab	44.8cd	0.42b	0.30ab	0.17ab
**P-BC**	400	36.8	58.4ab	1.8bc	3.5c	21.0c	38.0bc	0.54c	0.71bc	0.21b
	500	31.5	64.5c	1.7bc	2.8bc	18.5b	44.0c	0.43b	0.51b	0.17ab
	600	28.5	71.9de	1.6b	2.0b	15.0ab	52.4d	0.31a	0.33ab	0.13a
	700	25.8	74.4e	1.4ab	1.4a	14.2a	62.5de	0.29a	0.22a	0.11a
**A-W-BC**	500	89.6	72.3	1.4	3.0	18.6	59.8	0.39	0.49	0.15
**W-W-BC**	500	96.2	73.6	1.5	3.0	15.9	59.2	0.32	0.48	0.13
**A-C-BC**	500	64.9	72.6	2.4	3.3	17.8	35.6	0.37	0.55	0.15
**W-C-BC**	500	92.0	78.1	1.6	3.1	14.3	58.0	0.28	0.48	0.11
**A-P-BC**	500	63.3	83.9	1.5	1.7	10.4	64.4	0.19	0.25	0.08
**W-P-BC**	500	78.7	69.0	2.4	3.3	18.8	33.8	0.41	0.58	0.17

The biochars include wheat-straw biochar (W-BC), corn-straw biochar (C-BC) and peanut-shell biochar (P-BC) as well as biochars pyrolyzed at 500°C and washed with acid (A-W-BC, A-C-BC and A-P-BC) and deionized water (W-W-BC, W-C-BC and W-P-BC).

Note: Different letters indicate significant difference for the results in the same column, excluding the biochars washed with acid and water.

**Table 2:** pH values, electrical conductivity (EC), ash content, cation exchange capacity (CEC), BET surface area, pore volume and pore size of W-BC, C-BC and P-BC at different pyrolytic temperatures.

Biochars	Temp. (°C)	pH	EC (µs cm^−1^)	Ash content (%)	CEC (cmol kg^−1^)	Surface area (m^2^ g^−1^)	Pore volume (cm^3^ g^−1^)	Pore size (nm)
**W-BC**	400	8.2g	100j	11ed	4.0ef	10b	0.012ab	4.6bc
	500	8.3g	108i	11ed	5.1e	111ef	0.090c	3.3b
	600	9.2f	141k	12cd	1.3g	177f	0.110c	2.5a
	700	9.2f	172k	15b	0.5g	107e	0.058b	2.2a
**C-BC**	400	10.2b	350d	14bc	38.3b	4a	0.008a	8.1d
	500	10.4a	864c	17a	68.6a	6a	0.012ab	2.1a
	600	10.4a	1936b	18a	20.1c	7ab	0.012ab	6.3cd
	700	10.4a	2221a	18a	19.0c	3a	0.006a	8.2d
**P-BC**	400	9.3e	204g	9e	7.2fg	5a	0.007a	5.2c
	500	9.4e	221h	10ed	8.5d	28c	0.022ab	3.2b
	600	9.6d	242f	11ed	1.2g	185f	0.110c	2.4a
	700	9.9c	261e	12cd	0.3g	49d	0.033b	2.7ab

Note: Different letters indicate significant difference for the results in the same column.

The crude product of biomass pyrolysis includes biochar and ash. To investigate the possible effects of ash on the sorption properties, samples of ash-free biochar were prepared. Crude biochar produced at 500°C was suspended in either 1 mol L^−1^ H_2_SO_4_ or DI water at 1.5 g in 30 mL and agitated for 2 h in an ultrasound bath [19]. Then the suspension was pumping filtrated until the pH of the filtrate stabilized between two consecutive extractions (±pH 0.02), and the filter cake was oven-dried (70°C) to obtain the treated biochars for further adsorption experiments. The acid-washed biochar is denoted as A-W-BC500, A-C-BC500, A-P-BC500, while the DI water-washed biochar as W-W-BC500, W-C-BC500, W-P-BC500. The biochar production rate and chemical compositions after washing are listed in Table 1.

### Determination of physical and chemical properties of the biochars

The specific surface area and porous texture of biochar were determined from N_2_ adsorption isotherms at 77 K with a Surface Area and Porosity Analyzer (ASAP 2020 HD88, USA). Biochar samples were degassed under vacuum at 363 K for 1 h and at 623 K for another 3 h, before being filled with N_2_ gas at different vapor pressures. The N_2_ adsorbed per g biochar was plotted versus the relative vapor pressure (P/Po) of N_2_ ranging from 0.02 to 0.2, and the data was fitted to the Brunauer-Emmett-Teller equation (BET) by computer to calculate surface area [23]. Biochar shapes and surface physical morphology were examined using FE-SEM (SU8000, Hitachi, Tokyo, Japan) at 15 KeV. The X-ray powder diffraction (XRD) patterns were determined using a Macscience-M18XHF instrument (UK) with Cu-Ka radiation at 40 mA and 40 kV. The data was collected over a 2θ range of 10–90° using the Cu-Ka radiation at a scan rate of 2° min^−1^ [23]. The FT-IR spectra were recorded on Bruker Vertex 70 Fourier transform infrared spectrometer using the oven-dried KBr (at 105–110°C) pellet technique (1∶100). The total number of scans was 32 with the spectral resolution of 4 cm^−1^.

Elemental contents of C, N, hydrogen (H) and oxygen (O) were determined using the Elemental Analyzer (vario PYRO cube). Biochar pH was measured using a pH meter (Mettler Toledo Delta 320) and electrical conductivity (EC) was by an electrical conductivity meter (DDS-307A), both with biochar to DI water ratio of 1∶30 w/w, after stirring for 1.5 min and equilibration for 1 h. Ash was separated by placing biochar sample in a nickel crucible and it was heated at 700°C for 2 h under air [24]. The content of ash was calculated as: Ash content (%)  =  (M_Ash_/M_Biochar_) ×100, where M_Ash_ was the mass of ash and M_Biochar_ was the mass of biochar. The CEC of biochar was measured by a modified NH_4_^+^-acetate compulsory displacement method [25]. An amount of 0.2 g biochar was leached with 20 mL DI water for five times, and the contents of K^+^, Na^+^, Ca^2+^ and Mg^2+^ in the collective leachate were determined as the soluble base cations of the biochar. After this, the biochar sample was leached with 20 mL of 1 M Na^+^-acetate (pH 7) for five times to determine K^+^, Ca^2+^ and Mg^2+^ in the leachate as the exchangeable base cations. The biochar samples were then washed with 20 mL of ethanol for five times to remove the excessive Na^+^. Afterwards, the Na^+^ on the exchangeable sites of the biochar was displaced by 20 mL of 1 mol NH_4_^+^-acetate (pH 7) for five times, and CEC was calculated from the Na^+^ displaced by NH_4_^+^. The contents of K^+^ and Na^+^ in the leachate were determined by flame photometry, and Ca^2+^ and Mg^2+^ by atomic absorption spectrometry.

### Sorption experiments

To investigate the ability of biochars to adsorb NH_4_^+^-N and NO_3_^−^-N, adsorption experiments were conducted by adding biochar samples to water solutions with different concentrations of NH_4_^+^-N and NO_3_^−^-N. The same experimental procedure was used for each type of biochar (excluding those treated with acid and DI water) and N solution. Specifically, 0.2 g biochar was added to 50 mL NH_4_Cl (or KNO_3_) solutions with concentrations of 10, 30, 50, 70, 100, 150, 300 and 500 mg NH_4_^+^ (or NO_3_^−^) L^−1^, respectively. The mixture was then shaken in a thermostatic shaker at 25°C and 200 rpm for 24 h to achieve equilibrium. The supernatant was filtered and analyzed for concentrations of NH_4_^+^-N (or NO_3_^−^-N) by a Flow Injector Auto analyzer (Auto Analyzer 3, High Resolution Digital Colorimeter). For the biochar samples treated with acid and DI water, the adsorption experiments were conducted only in NH_4_Cl (or KNO_3_) solution with 50 mg NH_4_^+^ (or NO_3_^−^) L^−1^, while the other experimental procedures were the same as for the non-washed biochar samples. The experiment for each sample was run in triplicate. The amount of NH_4_^+^-N (or NO_3_^−^-N) adsorbed on biochar was calculated as the difference between the original NH_4_^+^-N (or NO_3_^−^-N) concentration and the remaining aqueous concentration at equilibrium. The amount of NH_4_^+^-N (or NO_3_^−^-N) adsorbed per unit mass of biochar was calculated as Eq.1 [26].
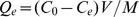
(1)where, Q_e_ is the amount of N adsorbed by biochars (mg g^−1^) at equilibrium; C_0_ and C_e_ are the NH_4_^+^-N (or NO_3_^−^-N) concentration in the initial and equilibrium solution (mg L^−1^), respectively; V is the volume of the aqueous solution (L) and M is the mass of biochar (g).

### Statistical analysis

The NH_4_^+^-N and NO_3_^−^-N sorption data were fitted to linear Freundlich and Langmuir models, which are the most frequently used models for describing sorption isotherms. The Freundlich adsorption model is as Eq. 2 [13]:
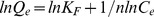
(2)where, Q_e_ is mass of NH_4_^+^-N or NO_3_^−^-N adsorbed per mass of biochar (mg g^−1^) at equilibrium; C_e_ is equilibrium concentration (mg L^−1^) of NH_4_^+^-N or NO_3_^−^-N in solution; K_F_ and 1/n are experimentally derived constants.

The Langmuir isotherm model, which assumes homogeneous monolayer surface sorption, can be written as Eq.3 [10]: 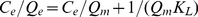
(3)where, Q_m_ is the maximum sorption capacity of biochar (mg g^−1^), and K_L_ refers to the Langmuir constants related to adsorption capacity and adsorption rate. When C_e_/Q_e_ is plotted against C_e_, a straight line with a slope of 1/Q_m_ and an intercept of 1/(Q_m_ K_L_) is obtained.

The results were expressed as means and standard deviations. Figures were plotted with the Origin 8.1 software. Statistical analysis was performed using Statistical Analysis System (SAS, version 9.1). Significant differences were tested using Duncan's multiple range test (*P* = 0.05) and the correlation was analyzed with the Pearson test (two-tailed) at *P* = 0.05. Any differences between the mean values at *P*<0.05 were considered statistically significant.

## Results and Discussion

### Yields and element contents of different biochars

The yields and element contents of biochars from wheat-straw, corn-straw and peanut-shell at four different pyrolysis temperatures of 400, 500, 600 and 700°C are given in Table 1. The yields of W-BC, C-BC and P-BC samples were reduced from 32.4%–36.8% to 22.8%–25.8% as pyrolysis temperature increased from 400 to 700°C. This is due to greater losses of volatile components at the higher pyrolysis temperatures [20].

Content of C, which is the major constituent of the biochars, increased with higher pyrolysis temperature for W-BC, C-BC and P-BC (Table 1). This was due to highly carbonization at high temperature (600°C and 700°C), with a high degree of C in aromatic structures [20]. However, contents of H and O decreased by approximately 60% and 30%, respectively, as pyrolysis temperature increased from 400°C to 700°C (Table 1). This was attributed to the removal of water, hydrocarbons, tarry vapors, H_2_, CO and CO_2_ during the carbonization process [26]. Some of these H and O contents are likely presented in organic functional groups on biochar surface [19]. Decrease of their contents is likely to result in a reduction in N sorption capacity. The biochar samples contained small amount of N (W-BC, 1.2–1.5%; C-BC, 1.6–2.4%; P-BC, 1.4–1.8%) and the N content remained relatively stable, which was consistent with the findings by Zheng et al. [23]. However, content of N in C-BC was always higher than that in W-BC and P-BC at a given temperature. This is most likely because corn straws had a much higher content of total N (17.2 g kg^−1^) than wheat straws (10.5 g kg^−1^) and peanut shells (12.4 g kg^−1^). Atomic ratios of elements, which estimates the aromaticity (H/C) and polarity (O/C, (O+N)/C) of the biochars, were significantly affected by pyrolysis temperature (Table 1). A higher H/C ratio shows a lower degree of carbonization and aromaticity of the biochar [19]. The atomic O/C ratios were also lower in W-BC700 (0.30), C-BC700 (0.42) and P-BC700 (0.29) than those in W-BC400 (0.56), C-BC400 (0.59) and P-BC400 (0.54), indicating the less hydrophilic surface of biochars at higher temperature [19].

Acid washing effectively removed most of the inorganic fractions from the three biochars (63.3–89.6%). Both acid and DI water washing affected the relative contents of the remaining elements in the biochars. Specifically, washing caused the proportion of C to increase, but not the proportions of H, N and O. Moreover, washing decreased the H/C and O/C atomic ratios in the biochars (Table 1).

### Characteristics of different biochars

#### 1. pH and EC values of biochars

All biochars produced in this study were alkaline, with a pH between 8.2 and 10.4 (Table 2). This range of pH is common for thermally produced biochars [1], [27]. In terms of different feedstocks, the pH values of W-BC (8.2–9.2) and P-BC (9.3–9.9) were lower than C-BC (10.2–10.4). The biochar pH significantly increased with higher pyrolysis temperature (*P*<0.05) (Table 2). For example, C-BC had a pH of 10.2 at 400°C and 10.4 at 700°C, which was consistent with the finding by Hossain et al. [28].

The biochars from the three feedstocks had a similar trend of EC values, that is, the values increased significantly with the higher pyrolysis temperature (Table 2). This apparent effect of pyrolysis temperature on EC values was consistent with the results of Cantrell et al. [29] and Quilliam et al. [30]. The EC estimates the amount of total dissolved salts or the total amount of dissolved ions in samples [10]. Its increase with pyrolysis temperature was likely due to loss of volatile materials at high temperatures, which promoted the relative concentrations of salts in the ash fraction.

#### 2. Ash contents and CEC of biochars

Ash contents in different biochars ranged from 11% to 18%, which were low compared with those in their feedstocks (wheat-straw 28%, corn-straw 31% and peanut-shell 27%). Apparently, ash content increased with rise in temperature due to increased concentrations of minerals and organic combustion residues [31]. Change of ash content in the biochars with temperature had a trend similar to that of biochars originated from other organic wastes such as pine needle and animal manure [32].

Biochar CEC values significantly differed with both feedstock and pyrolysis temperature. The CEC of C-BC (19.0–68.6 cmol kg^−1^) was much higher than that of P-BC (0.3–8.5 cmol kg^−1^) and W-BC (0.5–5.1 cmol kg^−1^), despite the fact that they had similar CEC in feedstocks (9.8–14 cmol kg^−1^). The trend of CEC changing with pyrolysis temperature was similar for the biochars from all feedstocks. All biochars pyrolysed at 400°C and 500°C had higher CEC than that at 600°C and 700°C. Whereas in the findings of Yuan et al. [24], CEC of biochar prepared from corn at 500°C was higher than that at 300°C and 700°C; and CEC of biochar prepared from peanut at 700°C was higher than that at 300°C and 500°C.

#### 3. Specific surface area and morphology structures of biochars

Specific surface area, pore volume and pore size of the biochars obtained from different feedstocks are summarized in Table 2. Biochar S_BET_ ranged from 3 to 185 m^2^ g^−1^, which was significantly affected by biochar feedstock and pyrolysis temperature [33]. In general, the S_BET_ of C-BC was much lower than that of W-BC and P-BC. Ahmad et al. [22] attributed this difference to the compositional compounds (lignin, cellulose and hemicellulose) in the original feedstocks, but the mechanisms behind were not clear. The S_BET_ of C-BC, W-BC and P-BC showed the same trend as affected by temperature, that is, S_BET_ increased as the temperature increased from 400 to 600°C, but substantially decreased at 700°C (Table 2). This is likely because of the removal of H- and O-carrying functional groups, including aliphatic alkyl-CH_2_, ester C = O, aromatic -CO and phenolic -OH groups, in biochars produced at 600°C, greatly enlarged their surface areas [32].

Pore structures of biochars as described by FE-SEM provide information about the structural change in biochar particles after thermal treatment. After pyrolysis, the biochars obtained rough surface and multiple sizes of pores, which resulted in a large specific surface area, a very important property for being sorbent materials [1]. FE-SEM micrographs of the morphological changes in the pore structure of the biochars at different temperatures and with different washing treatments implied that the clear and well-developed pore structure of the biochar consisted of cylinder-like tubes. The FE-SEM micrographs of C-BC as an example are shown in Figure S1a–f. The biochars contained microparticles and micropores, and the unregular fold structure changed into regular layer with the increasing temperature. But at 700°C, the biochars showed laminated texture. FE-SEM micrographs also demonstrated a homogeneous pore size distribution with a pore arrangement, and the pores in the inner portion of the biochars were obvious and well arranged in an array of cylinder-like structures. The above features of the FE-SEM micrographs, such as well-developed pore structure and pore size distribution, implied an excellent possibility for NH_4_^+^-N to be adsorbed by the biochars according to Sun et al. [34]. Compared with the non-washed C-BC500, biochar treated with diluted H_2_SO_4_ and DI water had some convexity structures (Figure S1e–f), and the pores increased after washing with acid.

#### 4. Crystal structure of biochars

Spectra for biochar crystal structure determined by XRD are shown in Figure S2a–c. Sharp peaks in all samples indicated presence of miscellaneous inorganic components, which suggested that there were quartz and sylvite in the biochar [24]. The XRD patterns for C-BC revealed sharp peaks, which showed a high degree of crystallinity with characteristic peaks at 26.6° (Figure S2b). The values matched the characteristic peaks of silicate carbonaceous (SiCO_3_^2−^) material, according to the database of the Joint Committee on Powder Diffraction Standards [34]. The XRD spectra analysis revealed that W-BCs (Figure S2a) and P-BCs (Figure S2c) had similar crystal substances as in C-BCs. Moreover, peak intensities decreased with higher temperature, indicating that inorganic components were well crystallized during low-temperature pyrolysis process [23]. However, the XRD spectra of different feedstocks at the same pyrolysis temperature showed no significant difference among the three types of biochars.

#### 5. Surface functional groups of biochars

**Figure 1:**
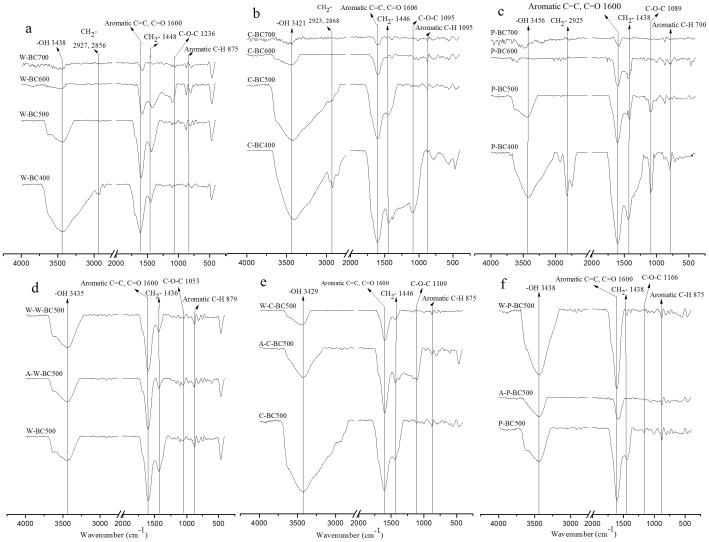
FT-IR spectrum of wheat-straw biochar (W-BC), corn-straw biochar (C-BC) and peanut-shell biochar (P-BC) at different pyrolytic temperatures and the biochars with different treatments at 500°C (a: W-BC, b: C-BC, c: P-BC, d: W-BC500 with acid- and DI water-washed treatments, e: C-BC500 with acid- and DI water-washed treatments, f: P-BC500 with acid- and DI water-washed treatments).

The FT-IR spectra of the twelve biochars are illustrated in Figure 1a–c. Different spectra reflected changes in the surface functional groups of biochars produced at different temperatures. The peak assignments in the spectra represented methyl C-H stretching compounds (∼2930 cm^−1^), methylene C-H stretching (∼2860 cm^−1^), aromatic carbonyl/carboxyl C = O (∼1700 cm^−1^), aromatic C = C and C = O (∼1600 cm^−1^), aliphatic C-O-C and alcohol-OH (1160–1030 cm^−1^), and aromatic C-H (∼815 cm^−1^) [18]. All these bands experienced different changes with increasing pyrolytic temperature, which is consistent with the study of Chen et al. [32]. At low pyrolysis temperatures (400–500°C) for W-BC, the band intensities were at 3438 cm^−1^ (-OH), which dramatically decreased and almost diminished at 600–700°C, whereas other bands (e.g., -CH_2_-, C = C and ester C = O) were preserved. The polar groups (-OH and C-O) exhibited the lower magnitude of peaks upon heating at high temperature (600°C and 700°C), suggesting a decrease in the polar functional groups with an increase in pyrolysis temperature. The maximum loss occurred in -OH, CH_2_- and C-O functional groups in biochars produced at 700°C, which was also apparent from their elemental compositions and element atomic ratio (Table 1). Thermal destruction of cellulose and lignin in the feedstocks might result in the exposure of aliphatic alkyl CH_2_-, hydroxyl -OH, ester C = O and aromatic C = O functional groups in biochars [32]. The changes in the peaks and their intensities and consequently functional groups of C-BC and P-BC were similar to those of W-BC. This is a result of strong dependence of the extent of carbonization on production temperature [31].

The comparisons of the functional groups between washed and non-washed biochars, as determined by FT-IR spectra, are presented in Figure 1d–f. After different treatments, the bands of W-BC, C-BC and P-BC changed considerably. FT-IR spectra confirmed that acid and DI water washing effectively removed most of the inorganic fractions of biochars. As seen from the FT-IR spectra in W-BC (Figure 1d), surface functional groups did not change by washing, which was supported by other studies [19], [32]. Compared with C-BC500 (Figure 1e), there were some differences in the FT-IR spectrum of A-C-BC500 and W-C-BC500. The strong peak at 1446 cm^−1^ (aromatic C = C) and 1600 cm^−1^ (-OH) decreased due to C condensation for A-C-BC500 and W-C-BC500 [35]. There was no peak at 1109 cm^−1^ (C-O-C) in C-BC500 and the new aromatic structure formed in A-C-BC500. But for P-BC (Figure 1f), the FT-IR spectra showed great difference. Compared with the non-washed samples, acid-washing decreased the intensities of surface functional groups at 3438 cm^−1^ (-OH) and 1600 cm^−1^ (aromatic C = C and C = O), but they were increased by DI water-washing. In addition, CH_2_- (1438 cm^−1^) diminished with acid- and DI water-washing.

### Ammonium nitrogen sorption on different types of biochars

**Figure 2:**
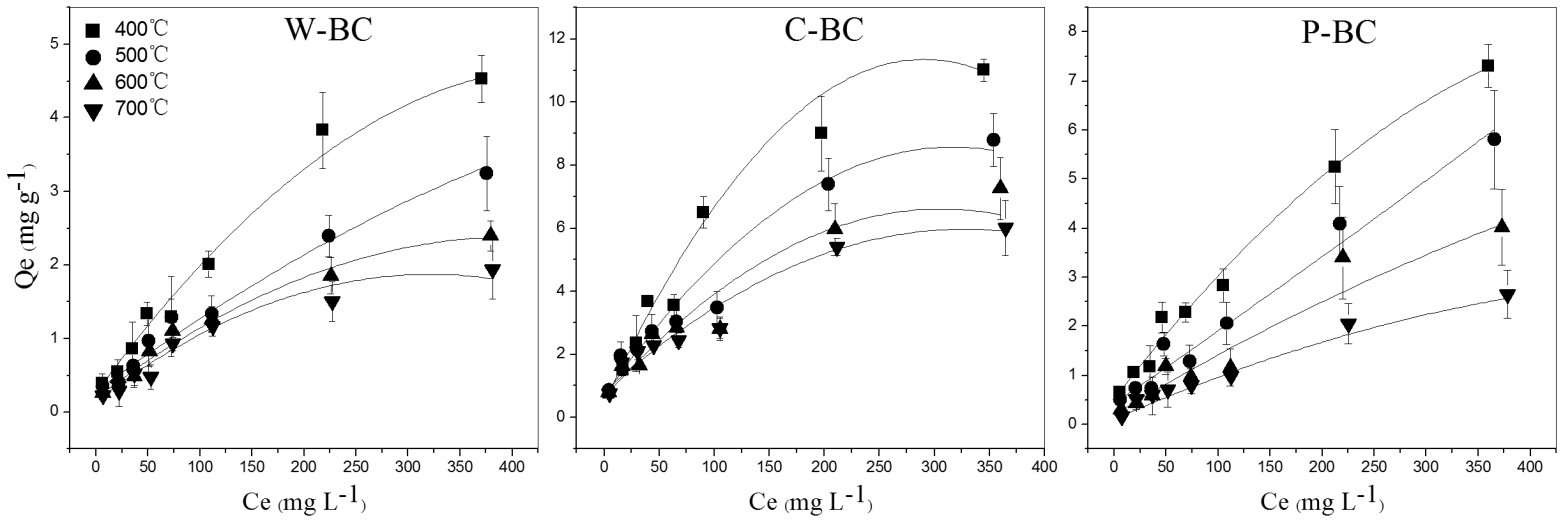
Sorption isotherms of NH_4_^+^-N on wheat-straw biochar (W-BC), corn-straw biochar (C-BC) and peanut-shell biochar (P-BC) at different pyrolytic temperatures (Q_e_: the amount of NH_4_^+^-N sorbed by per unit mass of biochar at equilibrium; Ce: concentration of NH_4_^+^-N in the solution at equilibrium). Bars indicate standard deviation of three replicates.

The equilibrium adsorption isotherms of NH_4_^+^-N, which are essential to understand the mechanism controlling biochar adsorption process, are presented in Figure 2a–c. The twelve tested biochars had considerable NH_4_^+^-N sorption capacity, e.g. 0.5–2.4 mg NH_4_^+^-N g^−1^ at an initial NH_4_^+^ concentration of 50 mg L^−1^. Biochars usually carry negative surface charges, which enhances the ability of soil to adsorb and retain cations (e.g. NH_4_^+^) and thus inhibit cation loss by leaching from acid soils [17], [20]. In general, C-BC had a greater NH_4_^+^-N sorption ability than W-BC and P-BC at a given pyrolysis temperature. For example, C-BC500 had a much higher Q_e_ value than W-BC500 and P-BC500, when the initial NH_4_^+^ concentration was 50 mg L^−1^ (Table 3). More NH_4_^+^-N was adsorbed by the low-temperature biochars (400–500°C) than by the high-temperature biochars (600–700°C) for each feedstock at a given NH_4_^+^-N concentration. Taking an initial concentration of 100 mg NH_4_^+^ L^−1^ as an example, C-BC400 (Q_e_ 3.6 mg g^−1^) and C-BC500 (Q_e_ 3.0 mg g^−1^) had relatively higher Q_e_ values than C-BC600 (2.8 mg g^−1^) and C-BC700 (2.4 mg g^−1^). Table 4 shows the Freundlich and Langmuir isotherm constants and NH_4_^+^-N adsorption correlation coefficients for different biochars. Sorption of NH_4_^+^-N to different biochars was better fitted to Freundlich isotherm model, with higher r values than that of Langmuir model. Despite both constants K_F_ and n in Freundlich model affect NH_4_^+^-N adsorption isotherms, it seems K_F_ plays a main role in reflecting differences of NH_4_^+^-N adsorption ability between biochars from different feedstocks. C-BC with a greater K_F_ value in the isotherm had a relatively high NH_4_^+^-N sorption ability compared with W-BC and P-BC. Compared with the non-washed biochars, washing with acid and DI water reduced adsorption of NH_4_^+^-N, especially for C-BC500 (Table 3). This decrease in NH_4_^+^-N sorption ability is probably because ash was washed off from the biochar and some functional groups were removed from the biochar surface, Zheng et al. [36] stated that ash could substantially improve theNH_4_^+^-N adsorption capacity of biochars.

**Table 3:** Sorption of NH_4_^+^-N and NO_3_^−^-N by W-BC500, C-BC500 and P-BC500 with different treatments in 50 mg L^−1^ aqueous solutions.

Biochars	Q_(NH4+)_ (mg g^−1^)			Q_(NO3-)_ (mg g^−1^)		
	Non-washed	Acid-washed	DI water-washed	Non-washed	Acid-washed	DI water-washed
**W-BC500**	0.63b	0.27a	0.33a	−0.25a	0.037bc	0.021b
**C-BC500**	2.12c	0.45b	0.92bc	−0.36a	0.058c	0.032bc
**P-BC500**	0.73b	0.43ab	0.54ab	−0.31a	0.042bc	0.024b

Note: Different letters indicate significant difference for the results and the adsorbed amounts of NH_4_^+^-N and NO_3_^−^-N were compared separately.

**Table 4:** Regression parameters of isotherms for expressing adsorption of solution NH_4_^+^-N to W-BC, C-BC and P-BC at different pyrolytic temperatures.

Biochars	Temp. (°C)	Freundlich model			Langmuir model		
		n	K_F_ (L mg^−1^)	R^2^	Q_m_ (mg g^−1^)	K_L_ (L mg^−1^)	R^2^
**W-BC**	400	1.5375	0.0954	0.9612	7.3314	0.0042	0.7709
	500	1.6717	0.0872	0.9398	4.6838	0.0050	0.8180
	600	1.7253	0.0789	0.9716	3.1636	0.0070	0.9191
	700	1.6949	0.0605	0.9437	2.6448	0.0065	0.8827
**C-BC**	400	1.5387	0.2778	0.9730	15.4560	0.0069	0.9117
	500	1.8212	0.3246	0.9540	12.0482	0.0065	0.7892
	600	2.1110	0.4188	0.9414	8.6201	0.0099	0.8291
	700	2.1711	0.3964	0.9611	7.1685	0.0114	0.8989
**P-BC**	400	1.6981	0.2019	0.9544	10.5153	0.0050	0.7561
	500	1.6139	0.1226	0.9048	9.9206	0.0032	0.4877
	600	1.4440	0.0604	0.9255	7.7761	0.0026	0.4132
	700	1.4821	0.0486	0.9681	4.01445	0.0044	0.7876

**Figure 3:**
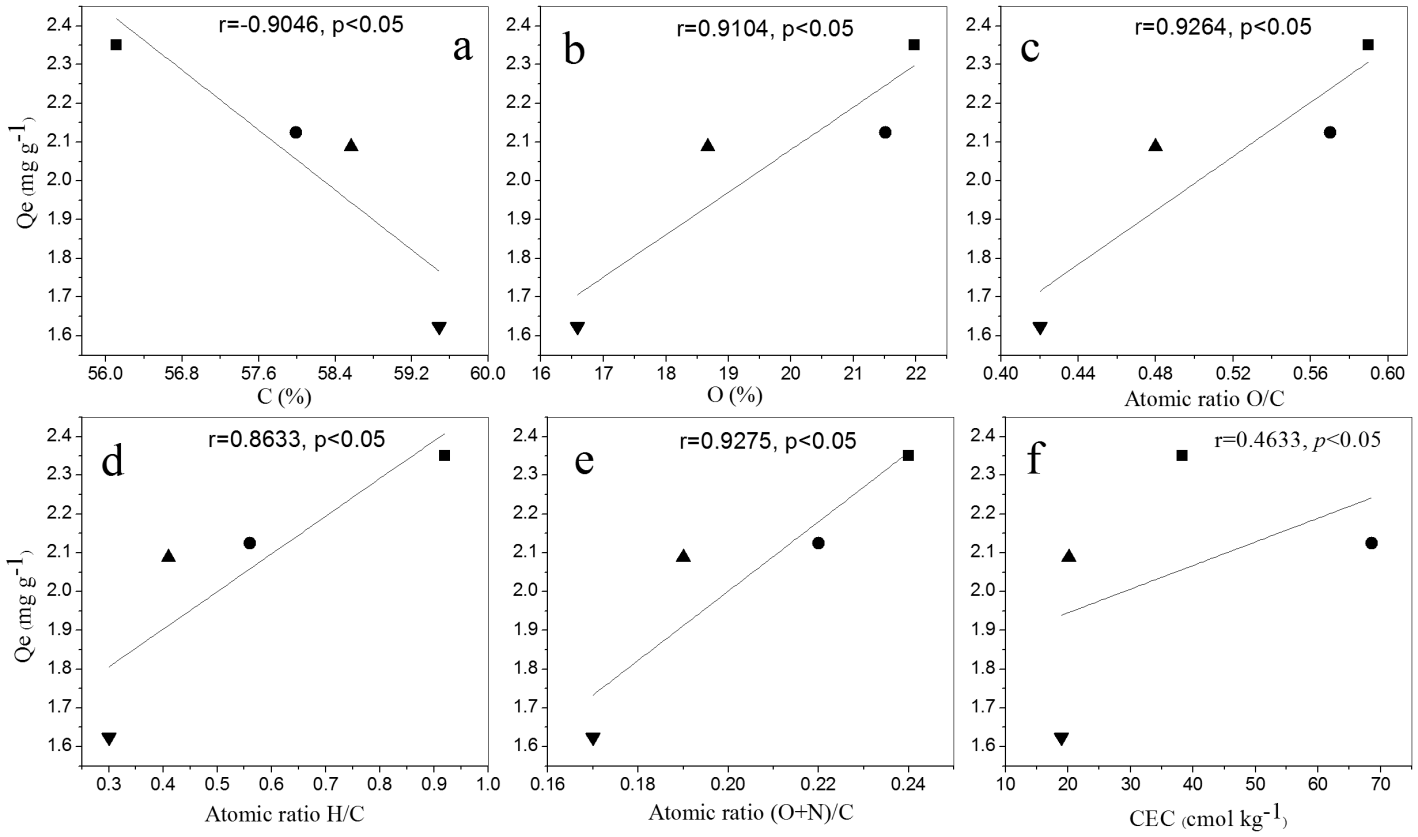
Correlations between mass of NH_4_^+^-N adsorbed per mass of biochar at equilibrium (Qe) and content of C (a), content of O (b), atomic ratio O/C (c), atomic ratio H/C (d), atomic ratio (O+N)/C (e) and CEC of corn-straw biochar (f), respectively. The symbols ▪, 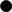, ▴ and ▾ represented pyrolysis temperatures at 400°C, 500°C, 600°C and 700°C, respectively.

To investigate the main factors influencing biochar ability to adsorb NH_4_^+^-N, correlations between Q_e_ and contents of elements and CEC of biochars were analyzed. The correlations for C-BCs as examples are shown in Figure 3a–f. The Q_e_ values were negatively correlated with C contents (r  = −0.9046) (Figure 3a). C-BC700 that had the highest C content among the C-BCs as a result of a high degree of carbonization at a high pyrolysis temperature [22], had the lowest adsorption amount of NH_4_^+^-N. In contrast, Q_e_ was positively correlated with content of O in the biochar (r = 0.9104) (Figure 3b). This indicated that removal of the O-carrying functional groups with increasing pyrolysis temperature induced the increase in the hydrophobicity of the BC600 and the BC700. As mentioned above, the biochars produced at high pyrolysis temperatures had low polarity (i.e. low O/C ratio) and thus low ability to adsorb NH_4_^+^. Positive correlations between Q_e_ and O/C (r = 0.9264), H/C (r = 0.8633) and (O+N)/C (r = 0.9275) were respectively observed (Figure 3c–e). All these implied a decrease in NH_4_^+^-N adsorption ability with decreasing polarity of biochars. In the present study, C-BCs had distinctly higher NH_4_^+^-N adsorption than W-BCs and P-BCs (Figure 2), despite the facts that C-BCs had obviously low specific surface area compared with the other biochars (Table 2). In addition, there was no clear trend of pore volume and pore size that could reflect the difference between biochars from different feedstocks, which was consistent with the finding by Yao et al. [14] in a test of thirteen biochars. These suggest that specific surface area and pore structures were not dominant factors affecting NH_4_^+^-N adsorption to biochars.

CEC seems to be the dominating factor influencing NH_4_^+^-N adsorption ability of biochars. The Q_e_ values were positively correlated with CEC (r = 0.4633) (Figure 3f). That is to say, the biochars with higher CEC values had larger NH_4_^+^-N sorption capacity. In the present study, C-BC had a higher adsorption capacity compared with W-BC and P-BC (Figure 2), which is most likely to be a result of the higher CEC values. For example, at 50 mg NH_4_^+^ L^−1^ solutions, the C-BC with CEC of 19.0–68.6 cmol kg^−1^ presented obviously higher NH_4_^+^-N sorption capacity (1.6–2.3 mg g^−1^) than the W-BC (0.6–0.9 mg g^−1^) and P-BC (0.6–1.2 mg g^−1^) with CEC of 0.3–8.5 cmol kg^−1^. In addition, biochars derived from each feedstock at pyrolysis temperatures of 600–700°C had relatively low CEC, compared with those at 400–500°C (Table 2). For instance, the CEC values of C-BCs decreased by 72% when the pyrolysis temperatures increased from 500 to 700°C. Correspondingly, NH_4_^+^-N adsorption ability of the biochars decreased with increasing pyrolysis temperatures. The decrease in CEC with increasing temperature can be attributed to the loss of carboxyl functional groups during pyrolysis [2].

### Nitrate nitrogen sorption on different types of biochars

**Figure 4:**
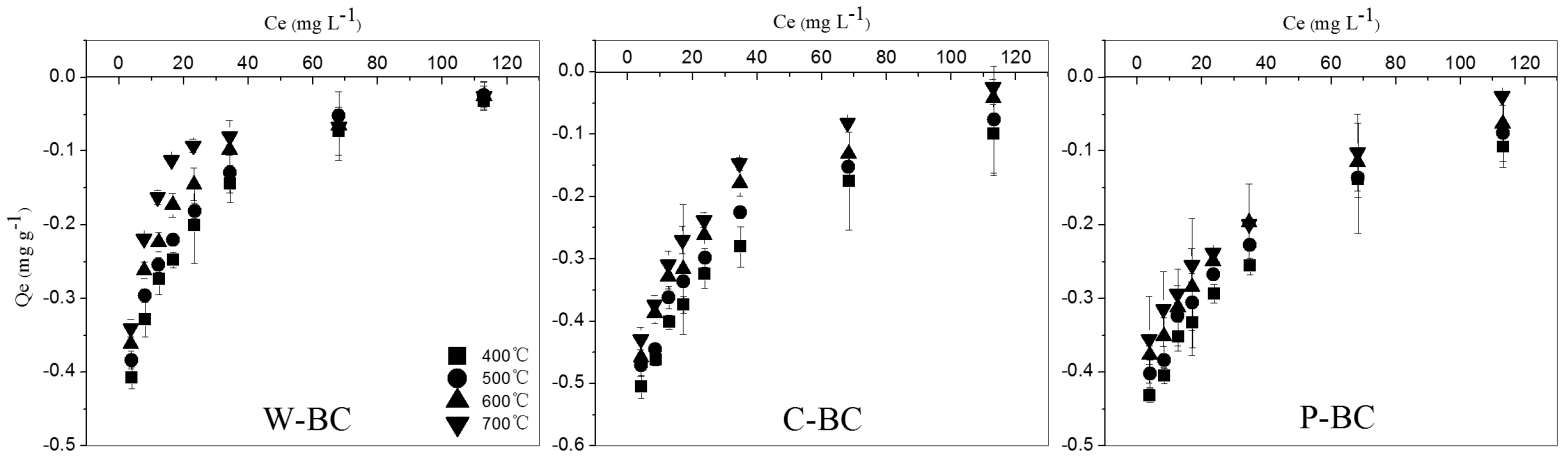
Sorption isotherms of NO_3_^−^-N on wheat-straw biochar (W-BC), corn-straw biochar (C-BC) and peanut-shell biochar (P-BC) at different pyrolytic temperatures (Q_e_: the amount of NO_3_^−^-N sorbed by per unit mass of biochar at equilibrium; Ce: concentration of NO_3_^−^-N in the solution at equilibrium). Bars indicate standard deviation of three replicates.

In contrast to NH_4_^+^, no NO_3_^−^-N could be sorbed by W-BC400-700, C-BC400-700 and P-BC400-700 at series of NO_3_^−^ concentrations (10–300 mg NO_3_^−^ L^−1^). On contrary, these biochars even released NO_3_^−^-N into the solutions (Figure 4a–c). Disability of biochars to adsorb NO_3_^−^-N was in agreement with the previous sorption experiments with the biochar made from sugarcane (*Saccharum officinarum* L.) bagasse (particle sizes 250–500 mm) at a temperature range of 400 to 600°C [37]. In the present study, the six-biochars made at a lower temperature (400–500°C) released 0.25–0.40 mg NO_3_^−^-N g^−1^ to the solution with an initial NO_3_^−^ concentration of 50 mg L^−1^ (Figure 4a–c). The other six biochars pyrolysed at higher temperatures released slightly less NO_3_^−^-N, at 0.16–0.32 mg g^−1^. Release of N in proportion of total N in biochar' ash was demonstrated with the following example. At an initial concentration of 10 mg NO_3_^−^ L^−1^, the N content in the ash of C-BC400 was 2.4% and an amount of 17 mg NO_3_^−^ was added with biochars to the 25 mL solution (Table 2). At equilibrium, the concentration of NO_3_^−^ in the solution was 18.95 mg L^−1^. That is to say, the N released accounted for 2.1% of the total N in the C-BC400. However, these results were opposite with the findings of previous studies, which reported that NO_3_^−^-N could be sorbed by biochars. For example, Mizuta et al. [38] reported that bamboo biochar powder (−80 mm) made at 900°C had NO_3_^−^-N adsorption capacity of 20.2 mg g^−1^ as estimated by the Langmuir model. A recent study by Hollister et al. [13] demonstrated that approximately 1.6 mg NO_3_^−^-N g^−1^ was adsorbed by the 800°C treated bagasse-biochar in the solution with an initial concentration of 20 mg NO_3_^−^-N L^−1^. Meanwhile, one study on NO_3_^−^-N sorption to bamboo-biochar (300–500 µm) gave a maximum sorption capacity of 7.1 mg NO_3_^−^-N g^−1^ predicted with the Langmuir adsorption model [39]. The weak ability of biochar to adsorb NO_3_^−^-N at different NO_3_^−^ concentrations in Mizuta et al. [38] and Hollister et al. [13] may be because their biochars were produced at a higher pyrolysis temperature (>800°C) than in the present study. Moreover, there may be other mechanisms involved to affect NO_3_^−^-N leaching in soil than direct adsorption of NO_3_^−^-N by biochars. Knowles et al. [40] found that biochar application reduced nitrate leaching from biosolid amended soils to levels at or below that in the control treatments in lysimeter experiments. Since we did not investigate the pyrolysis temperature higher than 700°C or N behavior in soil, further studies are needed in this regard to fully understand the mechanisms governing NO_3_^−^-N retention to biochars.

Washing with acid and DI water had a significant effect on biochar adsorption of NO_3_^−^-N (Table 3). In contrast to releasing of NO_3_^−^-N from the non-washed C-BC to solutions, small quantities of NO_3_^−^-N were adsorbed by A-C-BC (0.14 mg g^−1^) and W-C-BC (0.12 mg g^−1^) from 50 mg NO_3_^−^ L^−1^ solutions. This trend was similar for W-BC and for P-BC (Table 3). First of all, washing with acid and DI water removed ash from biochars, and thus no NO_3_^−^-N was added with the biochar ash to the solution. In addition, removal of the ash from biochars might have created additional sorption sites on biochar surface and facilitated more sorption of NO_3_^−^-N [41]. Afkhami et al. [42] suggested that treatment of biochars with acid tends to produce positive sites on the biochars, by protonation of surface -OH groups that would cause an increase in electrostatic adsorption of anions.

## Conclusions

Feedstock types and pyrolysis temperature greatly influenced the biochar chemical and physical characteristics, which further influenced N adsorption ability of the biochars. Adsorption of NH_4_^+^-N was predominantly affected by the CEC of biochars. The corn-straw biochar had the largest adsorption capacity for NH_4_^+^-N, in particular at a pyrolysis temperature of 400°C. In contrast, biochars released NO_3_^−^-N to the solutions rather than adsorb NO_3_^−^-N. However, retention of NO_3_^−^-N by biochar may be enhanced by promoting pyrolysis temperature or other mechanisms in soils. Therefore, we conclude that biochars, in particular corn-straw biochar (400°C), can be used under conditions where NH_4_^+^-N (or NH_3_) pollution is a concern, but further research is needed in terms of applying biochars to reduce NO_3_^−^-N pollution.

## Supporting Information

Figure S1 **Field emission-scanning electron microscopy (FE-SEM) images of biochars derived from corn straw pyrolytic at different temperatures and with different treatments (a: 400°C, b: 500°C, c: 600°C, d: 700°C, e: 500°C and treated with diluted H_2_SO_4_, f: 500°C and treated with DI water).**
(DOCX)

Figure S2 **The X-ray diffraction (XRD) spectrum of wheat-straw biochar (W-BC), corn-straw biochar (C-BC) and peanut-shell biochar (P-BC) at different pyrolytic temperatures.**
(DOCX)
