# Supplementary material for: Effects of Feedstock and Pyrolysis Temperature on Biochar Adsorption of Ammonium and Nitrate
Source: PLoS One. 2014 Dec 3;9(12):e113888. doi: 10.1371/journal.pone.0113888 (PMC4254611; doi:10.1371/journal.pone.0113888)
Supplement: Figure S1 — Field emission-scanning electron microscopy (FE-SEM) images of biochars derived from corn straw pyrolytic at different temperatures and with different treatments (a: 400°C, b: 500°C, c: 600°C, d: 700°C, e: 500°C and treated with diluted H2SO4, f: 500°C and treated with DI water). (DOCX) [file pone.0113888.s001.docx]

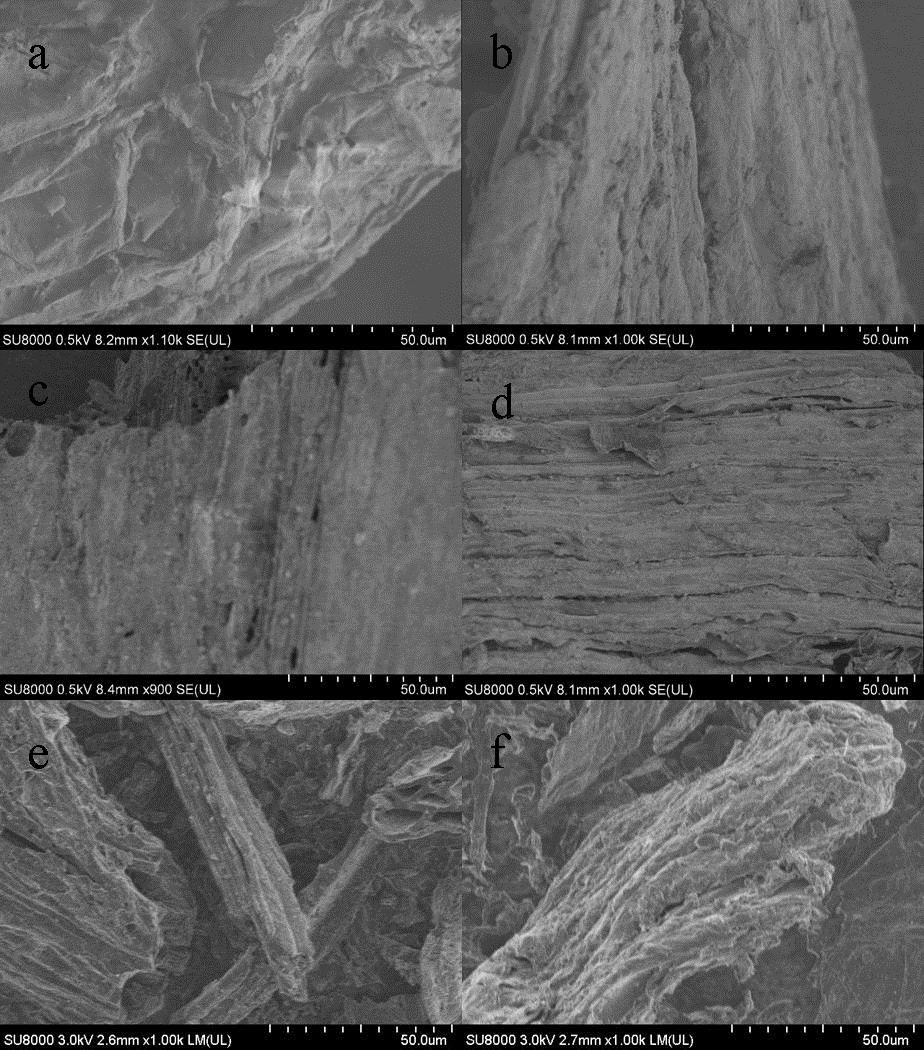


**Figure S1. Field emission-scanning electron microscopy (FE-SEM) images of biochars derived from corn straw pyrolytic at different temperatures and with different treatments (a: 400℃, b: 500℃, c: 600℃, d: 700℃, e: 500℃ and treated with diluted H_2_SO_4_ , f: 500℃ and treated with DI water).**
