# Supplementary material for: Effects of Feedstock and Pyrolysis Temperature on Biochar Adsorption of Ammonium and Nitrate
Source: PLoS One. 2014 Dec 3;9(12):e113888. doi: 10.1371/journal.pone.0113888 (PMC4254611; doi:10.1371/journal.pone.0113888)
Supplement: Figure S2 — The X-ray diffraction (XRD) spectrum of wheat-straw biochar (W-BC), corn-straw biochar (C-BC) and peanut-shell biochar (P-BC) at different pyrolytic temperatures. (DOCX) [file pone.0113888.s002.docx]

**Figure S2. The X-ray diffraction (XRD) spectrum of wheat-straw biochar (W-BC), corn-straw biochar (C-BC) and peanut-shell biochar (P-BC) at different pyrolytic temperatures.**
